# Industrial Fruits By-Products and Their Antioxidant Profile: Can They Be Exploited for Industrial Food Applications?

**DOI:** 10.3390/foods10020272

**Published:** 2021-01-29

**Authors:** Cássia H. Barbosa, Mariana A. Andrade, Raquel Séndon, Ana Sanches Silva, Fernando Ramos, Fernanda Vilarinho, Khaoula Khwaldia, Letricia Barbosa-Pereira

**Affiliations:** 1 Department of Food and Nutrition, National Institute of Health Dr. Ricardo Jorge, Av. Padre Cruz, 1649-016 Lisbon, Portugal; cassia.barbosa@insa.min-saude.pt (C.H.B.); mariana.andrade@insa.min-saude.pt (M.A.A.); fernanda.vilarinho@insa.min-saude.pt (F.V.); 2 Faculty of Pharmacy, University of Coimbra, REQUIMTE/LAQV, Coimbra, Azinhaga de Santa Comba, 3000-548 Coimbra, Portugal; 3 Analytical Chemistry, Nutrition and Food Science Department, Pharmacy Faculty, University of Santiago de Compostela, 15782 Santiago de Compostela, Spain; raquel.sendon@usc.es (R.S.); letricia.barbosa.pereira@usc.es (L.B.-P.); 4 National Institute for Agricultural and Veterinary Research (INIAV), I.P., Rua dos Lagidos, Lugar da Madalena, 4485-655 Vairão, Vila do Conde, Portugal; 5 Center for Study in Animal Science (CECA), ICETA, University of Oporto, 4051-501 Oporto, Portugal; 6 Laboratoire des Substances Naturelles, Institut National de Recherche et d’Analyse Physico-Chimique, INRAP, Pôle Technologique de Sidi Thabet, Sidi Thabet 2020, Tunisia; khaoula_khwaldia@yahoo.fr

**Keywords:** antioxidant capacity, apple, industrial by-products, LC-DAD, lemon, orange, UHPLC-ESI-MS/MS

## Abstract

Fruit by-products have a low economic value and have proven biological activities, such as antioxidant capacity due to the presence of active compounds. The main objective of this study was to obtain and determine the antioxidant capacity, through DPPH radical assay and β-carotene bleaching assay, of three food grade extracts from apple, lemon, and orange industrial by-products. Furthermore, the extracts were characterized by ultra-high performance liquid chromatography coupled to mass spectrometry (UHPLC-MS/MS). LC with diode array detector (LC-DAD) was used for the quantification of the main polyphenols. Lemon extract presented the highest inhibition percentage of DPPH radical (51.7%) and the highest total phenolics content (43.4 mg GAE/g) from the by-products studied. Orange by-product was that with the higher number of polyphenols while lemon extract was that with the highest content of individual phenolics. The by-product obtained from the lemon was that with higher amounts of hydroxycinnamic acids (407 µg/g of by-product), mainly chlorogenic acid (386.7 µg/g), followed by the apple by-product (128.0 µg/g of by-product), which showed higher amounts of rosmarinic and chlorogenic acids. These industrial by-products have great potential as a source of natural antioxidants to be used directly as food additives or to be incorporated in packaging to produce active food packaging.

## 1. Introduction

Fruits are often presented to consumers in several forms, such as jams, juices, concentrates, and pastes. The manufacturing processes of these formulations do not use the fruit entirely, originating a large quantity of fruit by-products that, in turn, have to be discarded in a responsible and sustainable way, which may imply a significant increase in the final price of the product [1,2]. Additionally, fruit by-products have a low economic value and have proven biological activities derived from the presence of phenolic compounds, vitamins, carotenoids, among other active compounds. Phenolic compounds, chemically characterized for having at least one phenol unit, are present in most terrestrial plants, and they are responsible for the plant’s defense against external stimuli such as radiation, predators and microorganisms [3,4]. The presence of these compounds is directly linked to the occurrence of antioxidant and antimicrobial activities, making these compounds of major interest for the food, cosmetic and pharmaceutical industries. 

Apple is a well-known fruit of the genus Malus (family Rosaceae) [5] and one of the most-consumed fruits all over the world [6]. According to the Food and Agriculture Organization of the United Nations, the global production of apples was over 85 million tons in 2019 [7]. Although the majority is consumed as a fresh fruit, 25–30% are converted into processed products, with apple juice being the main product [8]. Apples represent an important source of bioactive compounds like pectins, dietary fibers, vitamins, oligosaccharides, triterpenic acids and phenolic compounds, such as flavonols, monomeric and oligomeric flavanols, dihydrochalcones, anthocyanidins, *p*-hydroxycinnamic and *p*-hydroxybenzoic acids [5,6]. Apples with a higher content in phenolic compounds tend to have a higher antioxidant capacity. The content in phenolic compounds varies with edaphoclimatic conditions (such as weather and water availability), cultivation practices, harvesting, storage conditions, and apple cultivars, the apple cultivar being the main factor in determining the content on bioactive compounds [6,9]. Furthermore, differences can also be found among the different parts of the apple, since the peel contains a higher content in phenolic compounds than the flesh [5]. Apple pomace, the mixture of peel, core, seed, calyx, stem and soft tissue resulting from apple juice production, is the main by-product generated, accounting for close to 25% of the fresh apple weight [8,10] and has approximately 20–30% of dried matter [11]. 

Lemons and oranges are other well-known fruits, belonging to the genus *Citrus*, with a production of more than 95 million tones worldwide, in 2019 [7]. Lemon is mostly consumed as juice, originating a large quantity of lemon by-products, which are a very good source of dietary fiber, pectin, flavonoids, limonoids, coumarins and carotenoids [12]. Lemon essential oil can be obtained from lemon peels, which has proven antimicrobial activity against *Escherichia coli*, *Staphylococcus aureus* and *Pseudomonas aeruginosa* [13,14]. Oranges are also largely consumed in juice form, leaving a large trail of by-products. The orange peel, similar to lemon peel, is a good source of dietary fiber, pectin, phenolic acids and flavonoids, including polymethoxylated flavones and flavonols [15,16]. Dietary fiber is an important resource used in the prevention of cardiovascular diseases, diabetes, cancer and gastrointestinal disorders [17].

To delay the natural degradation of foods, the food industry resorts to antioxidant and antimicrobial additives, normally from a synthetic origin. The indirect and unaware consumption of these compounds has been associated with the promotion of carcinogenesis and their effects on human health due to long exposure are still unknown [2,18,19,20]. Therefore, it is important to find alternatives to these additives not associated with adverse health effects, such as extracts, and essential oils obtained from fruit by-products. Fruits are a good source of antioxidants, with important health benefits. Their by-products (peel, stems, and seeds) are also an excellent source of antioxidants [2,21]. However, there are not many studies on industrial fruit by-products. The majority of the studies are with specific parts of the by-products, such as peel, stems or seeds. Furthermore, there is not a method that can measure the antioxidant capacity precisely, therefore, different assays should be performed to obtain a more accurate result [22,23].

The main objective of this study was to obtain and determine three food-grade extracts from apple, lemon, and orange by-products and determine their antioxidant capacity. Moreover, the three extracts were chemically characterized, and their main compounds were quantified by UHPLC-ESI-MS/MS.

## 2. Materials and Methods

### 2.1. Fruits By-Product Extraction

The by-products of lemon, orange and apple were kindly supplied by the Portuguese juice company, Frubaça—Cooperativa de Hortofruticultores. Absolute ethanol was the chosen solvent for the production of the extracts since the main goal of the extract is to be applied directly or indirectly (through an active packaging) in foods. Ethanol is authorized by the Directive 2009/32/EC [24] in the extractions of bioactive compounds to be applied in foods. The samples were first grinded and freeze-dried, followed by the extraction process. Briefly, 5 g of sample 50 mL of absolute ethanol was added, the mixture was agitated on a compact shaker (Edmund Bühler GmbH model KS-15, Hechingen, Germany) at 450 rpm for 30 min at room temperature (23 ± 1 °C), protected from the light. Then, the mixture was centrifuged (Heraeus Multifuge X3 FR, Thermo Scientific, Langenbold, Germany) at 6000 rpm at 10 °C for 10 min. After that, the supernatant was removed to an amber pear-shaped flask and the ethanol was completely evaporated on a rotary evaporator (Büchi model R-210 Labortechnik, Switzerland) at 35 °C. The extract was removed with an aid of a spatula, held at −20 °C, protected from the light, until further use. To evaluate the antioxidant capacities of the different extracts, free radical DPPH inhibition and β-carotene bleaching assays were performed. In addition, the Total Phenolic Compounds (TPC) and the Total Flavonoid Content (TFC) were determined. To perform the antioxidant activity assays, the extracts obtained were dissolved in absolute ethanol, at a concentration of 3 mg/mL.

### 2.2. Antioxidant Activity

#### 2.2.1. Free Radical DPPH Inhibition Assay

For the free radical DPPH inhibition assay, the method described by Moure et al. (2001) [25] and modified by Andrade et al. (2018) [4], was applied. Briefly, 2 mL of a DPPH methanolic solution (14.2 μg/mL) were added to 50 μL of the sample. The mixture was homogenized and kept in the dark for 30 min, at room temperature (23 ± 1 °C). Absorbance was then measured at 515 nm using a spectrophotometer Evolution 300 UV-Vis (ThermoScientific™, England). A control assay was performed with the solvent in which the sample was dissolved. The inhibition percentage (IP) of DPPH was calculated according to the following Equation (1):

(1) $$\mathrm{IP}\left(\%\right)=\frac{\mathrm{Ac}-\mathrm{As}}{\mathrm{Ac}}\times 100$$

where Ac is the absorbance of the control and As is the absorbance of the sample.

Furthermore, a calibration curve using Trolox (6-hydroxy-2,5,7,8-tetramethylchroman-2-carboxylic acid) as a standard was drawn with a working range of 10–175 μg/mL.

#### 2.2.2. β-Carotene Bleaching Assay

The β-carotene bleaching assay was performed according to the described by Miller (1971) [26] and modified by Andrade et al. (2018) [4] Firstly, a solution with 20 mg of linoleic acid, 200 mg of Tween^®^40 and 1 mL of β-carotene in chloroform (0.2 mg/mL) was prepared. The chloroform was evaporated on a rotary evaporator at 40 °C. Then, 50 mL of MilliQ™ water was added, and vigorously agitated, until an emulsion was formed. Finally, to 200 µL of the sample, 5 mL of the emulsion was added. Afterwards, the absorbance of the control was measured at 470 nm and the mixtures were subjected to 50 °C for 120 min. The antioxidant activity coefficient (AAC) was calculated according to the Equation (2):

(2) $$\mathrm{AAC}=\frac{\left(A_{s120}-A_{c120}\right)}{\left(A_{c0}-A_{c120}\right)}\times 1000$$

where, A_s120_ is the absorbance of the sample after 120 min, A_c120_ is the absorbance of the control after 120 min and A_c0_ is the absorbance of the control at 0 min.

### 2.3. Total Phenolic Compounds Content (TPC)

The determination of the Total Phenolic Compounds Content was carried out according to the Erkan et al. (2008) [27] method. According to the method, 7.5 mL of an aqueous solution of Folin-Cioucalteu (10%, *v*/*v*) was added to 1 mL of sample. After 5 min, 7.5 mL of an aqueous solution of sodium carbonate (60 mg/mL, *w*/*v*) was added. Then, the samples were kept in the dark for 120 min, and the absorbance was measured at 725 nm. Gallic acid was used as a standard for the calibration curve, with a working range between 5–150 µg/mL. 

### 2.4. Total Flavonoid Compounds (TFC)

The Total Flavonoid Content method was performed according to the Yoo et al. (2008) [28] method. To 1 mL of sample, 4 mL of MilliQ water and 0.3 mL of aqueous solution of sodium nitrite (5%, *w*/*v*) were added, and the solution was homogenized. After 5 min, 0.6 mL of aqueous solution of aluminum chloride (10%, *w*/*v*) were added and the solution was once again homogenized. After 6 min, 2 mL of sodium hydroxide (1 M, *w*/*v*) and 2.1 mL of MilliQ™ water were added. The solution was homogenized and the absorbance was measured at 510 nm. Epicatequin was used as a standard for the calibration curve with a working range between 5–125 µg/mL. 

### 2.5. Identification of the Polyphenolic Compounds by UHPLC-ESI-MS/MS

The identification/tentative identification of phenolic compounds in the fruit by-products extracts was performed with a UHPLC-ESI-MS/MS (Thermo Fisher Scientific, San José, CA, USA), equipped with a degasser, Accela quaternary pump, autosampler, and column oven, coupled to a triple quadrupole mass spectrometer TSQ Quantum Access max. The instrument control and data collection and processing were performed with Xcalibur 2.1 software (Thermo Fisher Scientific, San José, CA, USA). 

A reverse-phase Kinetex^®^ EVO C18 100Å column (150 × 3 mm internal diameter, 5 μm particle size) (Phenomenex, Torrance, CA, USA) was used for phenolic compound separation at 30 °C, according to Andrade et al. [21] The injection volume was 20 μL, and the mobile phase flow rate used was 0.6 mL/min. The solvents used as mobile phase were water (solvent A) and methanol (solvent B), both acidified with formic acid at 0.1% (*v*/*v*). The gradient elution used was as follows: 95% A; 3 min, 90% A; 10 min, 80% A; 18 min, 70% A; 25 min, 30% A; 33 min, 0% A; 33–40 min, 0% A and 100% B isocratic; and finally, the column was washed and reconditioned with 95% A (40–46 min).

The mass spectrometer electrospray ionization source (ESI) operated in both negative and positive mode, according to the nature of the phenolic compound. The optimized MS/MS detector settings were as follows: spray voltage 2500 V; vaporizer and capillary temperatures were set at 340 °C and 350 °C, respectively. Nitrogen (purity > 99.98%) was used as sheath gas (pressure 35 psi) and as auxiliary gas (the pressure set 10 arbitrary units), and Argon as the collision gas (1.5 mTorr).

The MS/MS data acquisition was performed in a Single Reaction Monitoring (SRM) mode. After the first screening at MS scan range of 100–800 *m*/*z*, tentative identification of polyphenols was accomplished by comparing their precursor ion [M-H]^−1^ and mass spectrometry fragmentation pattern (MS/MS) with those already described in the literature. The identification of the individual phenolic compounds was accomplished by comparison of the retention time with those obtained by injecting pure standards, when available, under the same chromatographic conditions, and with the molecular ion and product-ions data provided by MS/MS analysis.

### 2.6. Quantification of the Polyphenolic Compounds by HPLC-DAD/UV

The quantification of phenolic compounds was performed with an Agilent HPLC system 1100 (Hewlett-Packard, Waldbronn, Germany), equipped with a quaternary pump, a degassing device, an autosampler, a column thermostat system, coupled to a diode array detector (DAD), and controlled by HP ChemStation software (version B.03.0.1). The column and chromatographic conditions used were the same described above for UHPLC-ESI-MS/MS analysis. DAD spectra acquisition was performed continuously in full scan modality during the run time ranging from 200 to 400 nm. The identification of individual phenolic compounds was achieved by comparing their retention times and the UV spectrum (λ_max_) characteristics of the different family of phenolic compounds or with that obtained with commercial standards injected under the same chromatographic conditions, whenever available. Phenolic compounds were monitored and quantified at 230, 278, 300, 325, and 360 nm. Quantification was carried out by the external-standard method with six-point calibration curves.

### 2.7. Statistical Analysis

All experiments were conducted using a completely randomized design with three replications. Statistical analysis of data was performed through a one-way analysis of variance (ANOVA) using the Software IBM^®^ SPSS^®^ Statistics, version 26.0.0.0, and differences among mean values were processed by the Tukey test. All requirements necessary to carry out the ANOVA (namely, normality of data and homogeneity of variances) have been validated. Significance was defined at *p* < 0.05. Results are expressed as the means of the replicants ± standard deviation.

## 3. Results and Discussion

### 3.1. Antioxidant Capacity

In this study, four assays were performed for a better characterization of the antioxidant. For antioxidant potential, DPPH radical scavenging capacity and β-carotene bleaching assay were performed. Besides that, TPC and TFC were quantified for antioxidant potential assessment. For all the assays, the extracts were analyzed in the concentration of 3 mg of extract per mL of ethanol.

Table 1 shows the IP of DPPH and the Trolox Equivalent (TE) for all the extracts. Lemon extract presented the highest IP of DPPH radical (51.67 ± 4.61%) followed by the apple extract (39.92 ± 1.68%) and orange extract (31.20 ± 1.28%). The DPPH radical scavenging capacity assay measures the reducing capacity of antioxidants.

Different results were obtained in other studies. Albuquerque et al. [29] evaluated a water extract obtained from industrial oranges by-products. The authors found lower values (898.9 µmol Trolox/L fruit by-products water extracts) when compared to the ethanolic extract of the orange by-products. This can be explained by the use of different extraction solvents in the two studies. Guimarães et al. [30] evaluated the antioxidant capacity of orange and lemon peel essential oils. The authors obtained good EC_50_ values for orange (95.67 ± 2.21 mg/mL) and lemon (116.25 ± 10.56 mg/mL). M'hiri et al. [12] studied the effects of different drying processes on the antioxidant activity of industrial lemon by-products. The authors concluded that all the drying processes decreased the total content of phenolic compounds, and antioxidant radical scavenging activity, supporting the room temperature extraction procedure of active compounds, such as the method used in the present study [12].

Regarding the β-carotene bleaching assay, orange extract (3 mg/mL) presented the highest AAC (237.21 ± 29.78) (Table 2). The β-carotene bleaching assay is also based on color change. In the absence of antioxidants, the free linoleic acid radical bonds to the β-carotene molecule and the orange color fade.

The lemon by-products extract presented the highest TPC (43.38 mg GAE/g ± 0.84 mg GAE/g) from the studied extracts. The TPC of orange and apple were 23.32 ± 0.18 mg GAE/g and 14.02 mg ± 0.13 mg GAE/g, respectively (Table 2). Phenolic compounds are recognized for their contribution as one of the most important antioxidants in the diet [29,31]. Therefore, it is essential to quantify the TPC presented in the food and its by-products. Guimarães et al. [30] analyzed lemon and orange peel and obtained a higher value of TPC, 87.77 mg/g extract and 79.75 mg/g, respectively. Li et al. [32] analyzed lemon and orange peel too, and the results presented as fresh matter were 118.75 mg/g and 73.59 mg/g, respectively. M’hiri et al. [12] analyzed lemon by-products and for TPC the results were 5.52 g/100 g as dry matter. On the other hand, the TPC obtained for apple by-products in this study were higher than the ones of Diñeiro García et al. [33]. Raudone et al. [6] quantified the TPC in apple by-products and the result was 31.01 mg/g as dry weight. It is also important to identify the individual phenolic compounds present in the fruits’ by-products.

The TFC of lemon, orange, and apple were 20.76 mg ± 0.61 mg ECE/g, 7.29 mg ± 0.32 mg ECE/g, and 24.63 mg ± 1.61 mg ECE/g (Table 2). Apple extract showed the highest TFC and orange extract showed the lowest TFC. Flavonoids are important phytonutrients too. The results obtained in this study for lemon by-products were higher than those from Guimarães et al. [30] M’hiri et al. [12] obtained 4.35 g/100 g as dry matter for TFC. For orange by-products, there were studies with higher and lower values than those obtained in this study [16,30]. No studies with TFC for apple by-products were found in the literature.

Dissimilarities in all results can be due to different fruits’ variability and their degrees of maturation. External factors such as climate, soil and fertilization applied can also affect the results. Apart from these, the results can be affected by the variability in the solvents used for the extractions and changes in the methods used [34,35,36,37].

In general, all three fruit by-products presented a good source of antioxidant compounds, able to be incorporated as dry extracts in food and in food packaging. However, from the studied industrial by-products, lemon extract was revealed to have the greatest potential as an antioxidant extract.

### 3.2. Chromatographic Polyphenolic Profile of the Fruit By-Products

**Figure 1:**
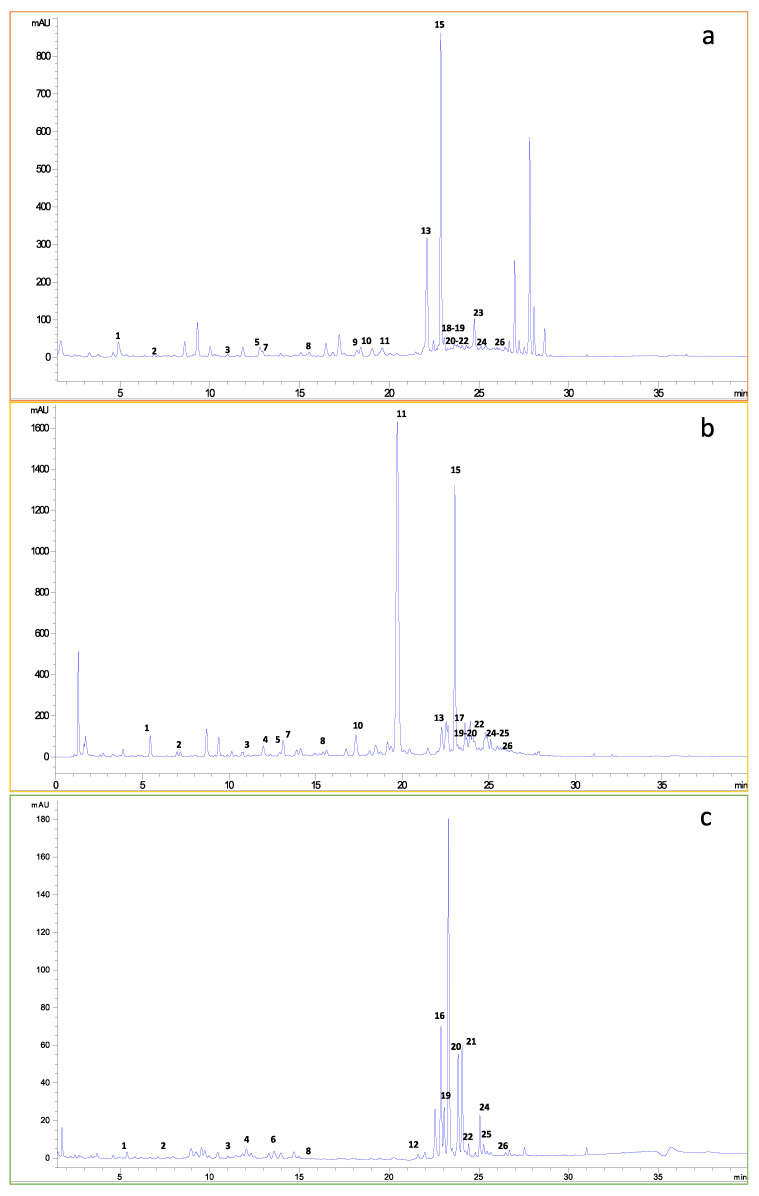
HPLC-DAD chromatograms of fruit by-products (**a**) orange, (**b**) lemon, and (**c**) apple, recorded at 278 nm.

The phenolic compounds of fruit by-products identified or tentatively identified by HPLD-DAD and UHPLC-ESI-MS/MS are described in Table 3. The identification of phenolic compounds was based on the elemental composition data determined from accurate mass measurements in negative ionization mode and comparison with the literature and that obtained with the available standards, except for compound 25 (Quercetin), which was measured in the positive mode as described previously by Andrade et al. [21] Each compound was characterized by its retention time (R_t_), maximum absorption wavelengths (λ_max_), structural class, molecular formula, molecular ion, and main MS/MS fragments. The peak names of the Table 3 correspond to the peak labels of the chromatograms obtained at 278 nm by HPLC–DAD for each fruit by-product represented in Figure 1. In this work, a total of 26 compounds (19 for orange, 18 for lemon, and 16 for apple by-products) from different classes of polyphenols were identified, including phenolic acids (benzoic acid derivates, hydroxycinnamic acids derivatives, and their glycosides) and flavonoids (flavonols, flavones, flavanones, and dihydrochalcones, as well as their glycosides). The confirmation of the identity of 17 polyphenols was achieved by a comparative analysis of authentic standards based on compounds retention time, the UV–visible spectra, and MS/MS fragmentation patterns.

#### 3.2.1. Benzoic Acid Derivates

The benzoic acid derivates identified in the fruit by-products analyzed were protocatechuic acid (compound 1) and hydroxybenzoic acid (compound 2) detected at UV λ_max_ 293 and 255 nm, respectively. The identification was performed by a comparison of their retention times and MS/MS fragmentation patterns with standards. The hydroxybenzoic acids are widely distributed in nature and have been identified by other authors in citrus and apple fruits and products [38,39,40]. On the other hand, protocatechuic acid has been described in apple fruits and less in citrus. Indeed, this study identifies for the first time protocatechuic acid in orange by-products.

#### 3.2.2. Hydroxycinnamic Acids and Their Glycosides

Several compounds from the group of hydroxycinnamic acids were identified in the fruit by-products by comparison of their retention times, UV–visible typical spectra at λ_max_ 325 nm, and MS/MS fragmentation patterns with standards. Caffeic and p-coumaric acids (compounds 3 and 8, respectively) were identified in all fruit by-products analyzed. Chlorogenic acid (compound 4) was found in lemon and apple by-products, while ferulic acid was determined just in orange by-products. Rosmarinic acid was found for the first time in orange by-products besides apple by-products [39]. Compound 5, with [M-H]^−^ ion at *m*/*z* 355 and the MS/MS fragment 193 *m*/*z* from ferulic acid, was tentatively identified as ferulic acid-*O*-hexoside. Additionally, compound 7, with [M-H]^−^ ion at *m*/*z* 385 and the MS/MS fragment 223 *m*/*z* from sinapic acid, was identified as sinapic acid-*O*-hexoside. These hydroxycinnamic acid glycosides were already described for orange pulp and juices by De Ancos et al. (2017) [41] Both compounds were considered for the first time for orange and lemon by-products in this work. 

#### 3.2.3. Flavanones Glycosides

Together with hydroxycinnamic acids, flavanone glycosides were the main group of phenolic compounds present in the by-products analyzed in this study, mainly in those obtained from citrus fruits.

Compound 24, with [M-H]^−^ ion at *m*/*z* 271, MS/MS fragment 151 *m*/*z*, and the UV–visible spectra typical at λ_max_ 290 nm, was identified as Naringenin in all by-products analyzed (orange, lemon, and apple). Besides, compound 11, with [M-H]^−^ ion at *m*/*z* 595 that displayed the same fragmentation pattern in negative ionization mode that results in the fragment 151 *m*/*z*, was identified as eriodyctiol-*O*-rutinoside (eriocitrin). For this compound, the UV–visible spectrum showed two λ_max_ at 290 and 330 nm, which are characteristic of flavanone glycosides and are usual to the following compounds identified in this group of phenolics. Naringenin-7-*O*-rutinoside (narirutin) (compound 13), with [M-H]^−^ ion at *m*/*z* 579, and naringenin-7-*O*-glucoside (prunin) (compound 20), with [M-H]^−^ ion at *m*/*z* 433, showed the same fragmentation pattern that results in the fragment 271 *m*/*z* of naringenin [40]. 

The MS/MS fragmentation of hesperidin (compound 15) and neohesperidin (compound 18), with the identical [M-H]^−^ ion at *m*/*z* 609, results in the same fragment 286 *m*/*z* and the UV–visible spectra λ_max_ at 290 and 355. On the other hand, compound 23 was tentatively identified as isosakuranetin-7-*O*-rutinoside (dydimin), with [M-H]^−^ ion at *m*/*z* 593, and MS/MS fragment 285 *m*/*z*, as described by De Ancos et al. 2017 [41] for orange.

The identity of compounds 11, 15, and 24 (eriocitrin, hesperidin, and naringenin, respectively) was confirmed by comparison with the retention time and fragmentation pattern of commercial standards. Naringenin and prunin were identified in all by-products analyzed, while neohesperidin and dydimin were found just in orange by-products. Eriocitrin, narirutin, and hesperidin were identified in citrus by-products (orange and lemon). 

The identification of phenolics, for which standards were not available, was supported by recent studies found in the literature on these groups of compounds described for citrus peels (orange and lemon) [41,42,43], besides some studies for apple products including peels [38,40,44].

#### 3.2.4. Flavonols and Flavonol Glycosides

The MS1 scan spectra, the UV–visible spectra typical at λ_max_ 270 nm and 360 nm, and the same fragmentation pattern in negative ionization mode that results in the fragment 301 *m*/*z* in negative ionization mode compared with those of authentic standards determined that compounds 17, 19, and 22 are flavonols glycosides. Quercetin-3-*O*-rutinoside (rutin) (compound 19) and quercitrin (compound 22) standards allowed the identification of these flavonols in all by-products analyzed (orange, lemon, and apple). These phenolics have been described in other studies for orange, lemon, and apple products/by-products [40]. Despite isoquercetin (compound 17) being described in apple fruit by Sommella et al. (2015) [44], in this study, it was just found in orange by-product and confirmed by the standard of reference. The identification of the aglycone quercetin (compound 25) in all fruit by-products was achieved by comparing the data with that obtained from the authentic standard. On the other hand, compound 14, with a [M-H]^−^ ion at *m*/*z* 593, was tentatively identified as kaempferol-3-*O*-rutinoside in orange and apple by-products based on the MS/MS fragment 285 *m*/*z*, the UV–visible spectra typical at λ_max_ 356 nm and supported by literature where this flavonol glycoside was described before for apple fruit [40].

#### 3.2.5. Others (Flavones and Glycosides, Dihydrochalcone Glycosides and Flavan-3-ols)

Compound 10 showed UV–visible spectra typical of flavones, and the MS1 spectra revealed a high intensity [M-H]^−^ ion at *m*/*z* 593. Moreover, the comparison of the relative absorbance at 270 and 340 nm allowed the identification flavone nature of the phenolic compound. Additionally, the fragment ion at *m*/*z* 473 described in the literature for di-*C*-glucoside flavanone confirmed the identification of this compound as apigenin-6,8-di-*C*-glucoside, more commonly identified as Vicenin-2. Apigenin-6,8-di-C-glucoside was previously identified in pulp and juices of orange and mandarins by De Ancos et al. (2017) [41], but was described for the first time in this study for orange and lemon by-products. Compound 26, with [M-H]^−^ ion at *m*/*z* 269, was identified as another flavone, the aglycone apigenin, that followed the same fragmentation pattern of the reference standard that results in the fragment 117 *m*/*z* in negative mode. This compound was described before for orange products such as pulp, juice, and peels [40,41]. Still, in this study, apigenin was detected for the by-products obtained from lemon and apple fruits. Phenolic compounds 12 and 16, with [M-H]^−^ ion at *m*/*z* 567 and [M-H]^−^ ion at *m*/*z* 435, respectively, followed the same fragmentation pattern that results in the fragment 273 *m*/*z*, were tentatively identified as dihydrochalcone glycosides. Compound 12 was, tentatively, identified as phloretin-*O*-apiofuranosyl-glucopyranoside, and compound 16 was identified as phloretin-2′-*O*-beta-glucoside (phlorizin) by comparison with the reference standard. These compounds have been described in the literature for apple pomace and were detected in the apple by-product analyzed in this study [40,45].

Finally, also exclusive for apple by-product, phenolic compound 6 with the [M-H]^−^ ion at *m*/*z* 289, the fragment ions at *m*/*z* 245 and 203, and the UV–visible spectra typical of flavan-3-ols at λ_max_ 278 nm, was identified as epicatechin and its identity confirmed with the commercial standard. Epicatechin was also already described in the literature for apple products [40,44].

### 3.3. Quantitative Distribution of Polyphenolic Compounds in Fruit By-Products

The quantification of phenolic compounds, for which the standards were available, was performed by HPLC-DAD following the parameters described in Table 4. The results on total and 15 individual phenolic compound contents are shown in Table 5. The total amount of phenolic compounds varied according to the nature of the by-product as follows: lemon (20,969 µg/g) < orange (5393 µg/g) < apple (894.8 µg/g). The results were in agreement with those obtained in the spectrophotometric assays of total phenolic compounds, even though several compounds present in the extracts were not identified and quantified (see Figure 1). The polyphenol content determined for lemon by-products using TPC assay was 2-fold higher than orange. However, according to the determination by HPLC-DAD, the lemon content was 4-fold higher than orange, since a substantial number of polyphenols in the orange extract were not able to be quantified. 

Benzoic acid derivates and flavanones glycosides were the most representative phenolics in the orange by-product, while for the lemon by-product, the most abundant were hydroxycinnamic acids, flavanone glycosides, and flavonol glycosides. The hydroxycinnamic acids and flavonol glycosides were the most relevant groups of compounds present in apple by-products, together with the flavanols glycosides and dihydrochalcone glycosides, which were exclusively present for this by-product.

The by-product obtained from lemon fruits was that with higher amounts of hydroxycinnamic acids (407 µg/g of by-product), manly chlorogenic acid (386.7 µg/g), followed by the apple by-product (128.0 µg/g), which showed higher amounts of rosmarinic acid (88.62 µg/g), besides chlorogenic acid (39.41 µg/g). These amounts in apple by-products could justify the highest antioxidant capacity of apple extract rather than the orange extract described in Section 3.1 for the DPPH assay. Similar contents of chlorogenic acid were determined in lemon peels by Xi et al. (2017) [46] in different lemon varieties, but not in other parts of the lemon fruit such as the pulp or juice that were more than 10-fold lower. On the other hand, the orange by-product was that with higher amounts of caffeic, ferulic, and p-coumaric acids, but the total amount (80.53 µg/g) was the lowest among the fruit by-products analyzed. These results were in agreement with those observed in the recent study on apple and orange peels, where the total amounts of phenolic acids, and in particular chlorogenic acid, were higher for the apple rather than orange peels [38]. Still, a high amount of protocatechuic acid (317.3 µg/g) was determined, in this study, for the orange by-product. These authors observed the same tendency for benzoic acid derivates such as hydroxybenzoic acid. However, the concentrations on orange and apple peels determined by these authors were higher than those observed in the by-products analyzed in this work.

Taking into account the flavanone glycosides as the most relevant group of compounds for citrus by-products, eriocitrin, hesperidin, and naringenin were those quantified. The compound present in high quantities in the orange by-product was the hesperidin (4901 µg/g), followed by naringenin, and finally Eriocitrin, in a total amount of 4956 µg/g. The content of hesperidin was up to 3-fold higher than those described by Molina-Calle et al. (2015) [42] for orange peels from different varieties in the range of 1200 and 1800 µg/g. De Ancos et al. (2017) [41] also determine these compounds for orange pulps and juices from different varieties (49–434 µg/g). Nevertheless, the results could not be compared with the literature since the authors express the results as fresh weight. However, the amounts of hesperidin were also significantly higher than eriocitrin for pulps and juices. On the other hand, the most abundant compound in lemon by-products was the eriocitrin (17,493 µg/g), followed by hesperidin (2728 µg/g), and finally naringenin (42.12 µg/g), in a total amount of flavanone glycosides of 20,263 µg/g. The amount of hesperidin was lower than that observed for the orange by-product. The amounts were lower than those described by Gómez-Mejía et al. (2019) [43], but these authors also observed that hesperidin was 2-fold higher in orange peels than in lemon peels.

Considering the flavonols glycosides, considerable amounts were determined for lemon by-product (262.3 µg/g), followed by apple (172.7 µg/g), and finally orange (39.40 µg/g). Isoquecetin and quercitrin were the most abundant in lemon with concentrations of 111.6 µg/g and 106.0 µg/g, respectively. The main compound in apple by-product was the quercitrin (150.3 µg/g), while in orange was the rutin (31.43 µg/g). Similar results for the rutin ratio among citrus samples (lemon and orange) have been described in the literature. However, the amounts depended on the variety or part of the fruit analyzed [43,46]. The amounts of quercitrin in apple by-product (150.3 µg/g) were more than 12-fold higher than those determined by Li et al. (2019) [45] in 7 varieties of apple flesh (2.7–12.4 µg/g). These authors also determined the epicatechin content for all varieties, and the values ranged from 5.8 to 80.7 µg/g. The amount of epicatechin in the apple by-product analyzed in this study was in the range with a concentration of 31.24 µg/g. This concentration of epicatechin could be related to the high values of TFC described for apple by-products in Section 3.1 and might display a higher response to this assay. These results highlighted that the chromatographic analysis should be considered to complement and achieve a correct characterization of extracts µg/g and therefore avoid the potential lack of specificity of spectrophotometric assays. 

Finally, phlorizin was the main phenolic compound quantified in apple by-product (542.0 µg/g), significantly higher than those observed in the apple fruit (11.4–40.9 µg/g). These compounds also may be related to the high antioxidant capacity of the apple by-product on the DPPH assay.

Since the phenolic composition of the fruits and their products may change with the variety and these by-products obtained from the food industry may result from a mix of varieties, predicting the content of polyphenols and their antioxidant potential could be a complex task. Therefore, a complete characterization of the by-products by chromatography is mandatory before their application as a food ingredient or additive.

## 4. Conclusions

The phenolic profile and the content of polyphenols of orange, lemon, and apple fruit by-products were determined by UHPLC-ESI–MS/MS and HPLC-DAD. This methodology may be employed for the routine screening of fruit by-products and the identification and quantification of polyphenols. The phenolic compounds responsible for the high antioxidant activity of citrus by-products, in particular for lemon, were hydroxycinnamic acids, flavonols glycosides, and flavanone glycosides. Eriocitrin was the main phenolic compound (17.49 mg/g) determined in lemon by-products, while for orange by-products it was hesperidin (4.9 mg/g). On the other hand, the antioxidant capacity of the apple byproduct could be due to the high content in hydroxycinnamic acids (e.g., rosmarinic acid) and other specific compounds such as epicatechin and phlorizin. 

The results encourage the valorization of the fruit by-products as powerful sources of natural antioxidants to be used as food additives or ingredients to increase the shelf life of foods and develop functional foods and active packaging, with potential health benefits creating new food market perspectives within the concept of a circular economy. 

## Figures and Tables

**Table 1:** DPPH radical scavenging capacity of different fruit by-products. The results are expressed as mean of three replicas ± SD. Different letters indicate statistical differences.

Fruits By-Products	Inhibition Percentage (%)	Trolox Equivalent (mg Trolox/g of Extract)
Lemon	51.67 ± 4.61 ^a^	33.17 ± 2.94 ^d^
Orange	31.20 ± 1.28 ^b^	20.13 ± 0.43 ^e^
Apple	39.92 ±1.68 ^c^	25.69 ± 0.56 ^f^

**Table 2:** Antioxidant capacity and characterization of different fruit by-products. The results are expressed as Mean ± SD. Different letters indicate statistical differences.

	β-Carotene Bleaching Assay	TPC (mg GAE/g)	TFC (mg ECE/g)
	Mean ± SD	Mean ± SD	Mean ± SD
Lemon	67.35 ± 1.96 ^a^	43.38 ± 0.84 ^c^	20.76 ± 0.61 ^f^
Orange	237.21 ± 29.78 ^b^	23.32 ± 0.18 ^d^	7.29 ± 0.32 ^g^
Apple	107.44 ± 23.81 ^a^	14.02 ± 0.13 ^e^	24.63 ± 1.61 ^h^

**Table 3:** Phenolic compounds identified in the fruit by-products from orange, lemon and apple by HPLC-DAD and UHPLC-ESI-MS/MS.

Peak	R_t_ (min)	λ_max_	[M-H] (*m*/*z*)	Main MS/MS Fragments (*m*/*z*)	Molecular Formula	Structural Class	Tentative Identification	By-Product			Confirmation/ Ref. ^§^
								Orange	Lemon	Apple	
1	4.31	260, 293	153	109, 108	C_7_H_6_O_4_	Benzoic acid derivates	Protocatechuic acid	✓	✓	✓	[38,39], Std*
2	7.09	255	137	93, 65	C_7_H_6_O_3_	Benzoic acid derivates	Hydroxybenzoic acid	✓	✓	✓	[38,39,40,41], Std*
3	9.59	325	179	134, 135	C_9_H_8_O_4_	Hydroxycinnamic acids	Caffeic acid	✓	✓	✓	[39,40,46], Std*
4	9.99	250, 325	353	191, 173	C_16_H_18_O_9_	Hydroxycinnamic acids	Chlorogenic acid		✓	✓	[38,39,40], Std*
5	11.42	240, 330	355	193	C_16_H_20_O_9_	Hydroxycinnamic acids glycosides	Ferulic acid-*O*-hexoside	✓	✓		[41]
6	11.64	280	289	245, 203	C_15_H_14_O_6_	Flavan-3-ols	Epicatechin			✓	[40,44], Std*
7	11.8	270, 330	385	223	C_17_H_22_O_10_	Hydroxycinnamic acids glycosides	Sinapic acid-*O*-hexoside	✓	✓		[41]
8	13.39	310	163	119, 93	C_9_H_8_O_3_	Hydroxycinnamic acids	p-Coumaric acid	✓	✓	✓	[38,39,40,46], Std*
9	15.26	325	193	134	C_10_H_10_O_4_	Hydroxycinnamic acids	Ferulic acid	✓			[38,39,40,46], Std*
10	15.68	270, 340	593	473	C_26_H_28_O_14_	Flavone glycosides	Apigenin-6,8-di-*C*-glucoside (Vicenin-2)	✓	✓		[41]
11	17.95	285, 330	595	287, 151, 135	C_27_H_32_O_15_	Flavanone glycosides	Eriodyctiol-*O*-rutinoside (Eriocitrin)	✓	✓		[41,46,47], Std*
12	20.29	270, 350	567	273	C_26_H_31_O_14_	Dihydrochalcone glycosides	Phloretin-*O*-apiofuranosyl-glucopyranoside			✓	[40]
13	20.56	290, 330	579	271	C_27_H_32_O_14_	Flavanone glycosides	Naringenin-7-*O*-rutinoside (Narirutin)	✓	✓		[40,41,42]
14	21.00	356	593	285	C_27_H_30_O_15_	Flavonol glycosides	Kaempferol-3-*O*-rutinoside	✓		✓	[40]
15	21.36	290, 355	609	300, 286, 242	C_28_H_34_O_15_	Flavanone glycosides	Hesperetin-7-*O*-rutinoside (Hesperidin)	✓	✓		[40,41,42,43,46,47], Std*
16	21.37	278	435	273, 167, 123	C_21_H_24_O_10_	Dihydrochalcone glycosides	Phloretin-2′-*O*-beta-glucoside (Phlorizin)			✓	[40], Std*
17	21.49	360	463	301, 271	C_21_H_20_O_12_	Flavonol glycosides	Quercetin-3-*O*-glucoside (Isoquercetin)		✓		[38], Std*
18	21.52	290, 355	609	286	C_28_H_34_O_15_	Flavanone glycosides	Hesperetin-7-*O*-neohesperidoside (Neohesperidin)	✓			[42]
19	21.58	255, 360	609	300, 271	C_27_H_30_O_16_	Flavonol glycosides	Quercetin-3-*O*-rutinoside (Rutin)	✓	✓	✓	[40,41,43,44,46,47], Std*
20	22.28	270, 350	433	301, 271	C_21_H_22_O_10_	Flavanone glycosides	Naringenin-7-*O*-glucoside (Prunin)	✓	✓	✓	[40]
21	22.49	250, 330	359	197, 161	C_18_H_16_O_8_	Hydroxycinnamic acids	Rosmarinic acid	✓		✓	[39], Std*
22	22.51	250, 350	447	331, 300, 301	C_21_H_20_O_11_	Flavonol glycosides	Quercetin-3-*O*-rhamnoside (Quercitrin)	✓	✓	✓	[38,40,44,45], Std*
23	23.19	285, 330	593	285	C_28_H_34_O_14_	Flavanone glycosides	Isosakuranetin-7-*O*-rutinoside (Dydimin)	✓			[41]
24	23.71	295	271	151, 119	C_15_H_12_O_5_	Flavanone	Naringenin	✓	✓	✓	[40,41,42,44,46], Std*
25	23.84	270, 360	274	70, 88	C_15_H_10_O_7_	Flavonols	Quercetin	✓	✓	✓	[21,38,40,41,47], Std*
26	24.9	280, 360	269	117	C_15_H_10_O_5_	Flavones	Apigenin		✓	✓	Std*

R_t_—retention time; λ_max_—maximum absorption wavelengths; [M–H]^−^—molecular ions; ✓—indicates the presence of the compound identified; **^§^** Ref—references used to support tentative identification of compounds and Std* (standards available) to confirm the identification.

**Table 4:** Analytical parameters of detection maximum absorption wavelength (λ_max_), linearity, LOD, and LOQ employed to determine bioactive phenolic compounds studied.

Phenolic Compound	Detection λ_max_ (nm)	Slope	Intercept	R^2^	Concentration Range (µg/mL)	LOD (µg/g By-Product)	LOQ (µg/g By-Product)
Protocatechuic acid	300	40.63	−4.334	0.9980	0.05–20	0.04	0.10
Hydroxybenzoic acid	278	210.0	−14.52	0.9990	0.10–20	0.10	0.20
Caffeic acid	325	146.6	−40.50	0.9980	0.10–20	0.10	0.21
Chlorogenic acid	325	61.58	−14.27	0.9989	0.10–20	0.10	0.20
Epicatechin	278	21.66	−2.006	0.9996	0.10–20	0.10	0.20
*p*-Coumaric acid	325	169.6	−10.05	0.9990	0.05–20	0.04	0.10
Ferulic acid	325	159.9	1.128	0.9999	0.05–20	0.05	0.20
Eriocitrin	278	19.12	−1.025	0.9999	0.05–20	0.04	0.10
Hesperidin	278	50.42	−2.975	0.9997	0.03–20	0.01	0.05
Phlorizin	278	24.78	0.1248	0.9999	0.05–20	0.04	0.10
Isoquercitrin	360	52.55	5.218	0.9994	0.05–20	0.04	0.10
Rutin	360	49.90	−3.978	0.9999	0.05–20	0.04	0.10
Rosmarinic acid	325	117.5	−21.47	0.9987	0.05–20	0.04	0.10
Quercetrin	360	67.76	−4.616	0.9998	0.05–20	0.05	0.10
Naringenin	300	70.09	−22.15	0.9970	0.05–20	0.03	0.05

R^2^—Coefficient of determination; LOD—Limit of determination; LOQ—Limit of quantification.

**Table 5:** Total and individual phenolic compounds contents (µg/g dry basis) in the by-products from orange, lemon and apple fruits determined by HPLC-DAD.

Phenolic Compound (µg/g of By-Product)	Fruit By-Product		
	Orange	Lemon	Apple
**Benzoic acid derivates**			
Protocatechuic acid	317.3 ± 7.173	16.08 ± 1.729	2.465 ± 0.0248
Hydroxybenzoic acid	<LOQ	20.27 ± 1.053	1.939 ± 0.0472
**Σ**	**317.3 ± 7.173**	**36.35 ± 0.7208**	**4.404 ± 0.0682**
**Hydroxycinnamic acids**			
Caffeic acid	26.19 ± 1.195	14.67 ± 0.4107	<LOQ
Chlorogenic acid	n.a.	386.7 ± 11.80	39.41 ± 1.016
*p*-Coumaric acid	18.49 ± 0.5547	6.424 ± 0.2468	<LOQ
Ferulic acid	22.88 ± 0.9469	n.a.	n.a.
Rosmarinic acid	12.97 ± 0.6995	n.a.	88.62 ± 3.606
**Σ**	**80.53 ± 2.915**	**407.8 ± 12.17**	**128.0 ± 4.554**
**Flavan-3-ols**			
Epicatechin	n.a.	n.a.	31.24 ± 0.7253
**Σ**			**31.24 ± 0.7253**
**Flavanone glycosides**			
Eriocitrin	24.63 ± 1.409	17,493 ± 115.5	n.a.
Hesperidin	4901 ± 155.4	2728 ± 17.32	n.a.
Naringenin	30.09 ± 0.4647	42.12 ± 0.8605	16.33 ± 0.834
**Σ**	**4956 ± 156.9**	**20,263 ± 131.6**	**16.33 ± 0.834**
**Flavonols glycosides**			
Isoquercetin	n.a.	111.6 ± 0.8220	n.a.
Rutin	31.43 ± 1.130	44.72 ± 0.5788	22.44 ± 0.977
Quercetrin	7.964 ± 0.4449	106.0 ± 0.3979	150.3 ± 3.769
**Σ**	**39.40 ± 0.9735**	**262.3 ± 1.793**	**172.7 ± 4.709**
**Dihydrochalcone** **glycosides**			
Phlorizin	n.a.	n.a.	542.0 ± 7.882
**Σ**			**542.0 ± 7.882**
**Total content (µg/g)**	**5393 ± 166.1**	**20,969 ± 144.7**	**894.8 ± 16.22**

Results expressed as mean values (*n* = 3) ± standard deviation. n.a. Not applicable. The summation (Σ) of each class of phenolic compounds is highlighted in bold.

## Data Availability

No new data were created or analyzed in this study. Data sharing is not applicable to this article.
